# Training for Transformation: Opportunities and Challenges for Health Workforce Sustainability in Developing a Remote Clinical Training Platform

**DOI:** 10.3389/fpubh.2021.601026

**Published:** 2021-04-20

**Authors:** Jana Muller, Cameron Reardon, Susan Hanekom, Juanita Bester, Francois Coetzee, Kopano Dube, Elmarize du Plessis, Ian Couper

**Affiliations:** ^1^Ukwanda Centre for Rural Health, Faculty of Medicine and Health Sciences, Stellenbosch University, Cape Town, South Africa; ^2^Physiotherapy, Faculty of Medicine and Health Sciences, Stellenbosch University, Cape Town, South Africa; ^3^Occupational Therapy, Faculty of Medicine and Health Sciences, Stellenbosch University, Cape Town, South Africa; ^4^Dr. Harry Surtie Hospital, Northern Cape Department of Health, Upington, South Africa

**Keywords:** rural, undergraduate, clinical training, distance education, workforce sustainability

## Abstract

**Background:** In 2018, Stellenbosch University's Ukwanda Centre for Rural Health led a faculty initiative to expand undergraduate health professions training to a new site, 9 hours drive from the health sciences campus in the sparsely populated Northern Cape Province of South Africa in the town of Upington. This is part of a faculty strategy to extend undergraduate health sciences training into an under-resourced part of the country, where there is no medical school. During 2019, the first year of implementation, four final year medical students undertook a longitudinal integrated clerkship at this site, while final year students from other programmes undertook short 5-week rotations, with plans for extending rotations and including more disciplines in 2020. The aim of this study was to understand stakeholder perceptions regarding the development of Upington as a rural clinical training site and how this influenced existing services, workforce sustainability and health professions education.

**Methods:** An iterative thematic analysis of qualitative data collected from 55 participants between January and November 2019 was conducted as part of the case study. A constructivist approach to data collection was utilized to explore participants' perceptions, experiences and understanding of the new training site. Triangulation of data collection and reflexive thematic analysis contributed to the trustworthiness of the data and credibility of the findings.

**Findings:** The perceptions of three key groups of stakeholders are reported: (1) Dr. Harry Surtie Hospital and Academic Programme Managers; (2) Supervising and non-supervising clinical staff and (3) Students from three undergraduate programs of the Faculty. Five themes emerged regarding the development of the site. The themes include the process of development; the influence on the health service; workforce sustainability; a change in perspective and equipping a future workforce.

**Discussion:** This case study provides data to support the value of establishing a rural clinical training platform in a resource constrained environment. The influence of the expansion initiative on the current workforce speaks to the potential for improved capacity and competence in patient management with an impact on encouraging a rural oriented workforce. Using this case study to explore how the establishment of a new rural clinical training site is perceived to influence rural workforce sustainability and pathways, may have relevance to other institutions in similar settings. The degree of sustainability of the clinical training initiative is explored.

## Introduction

Rural clinical exposure during undergraduate health professions training is one of a number of important educational strategies to develop and strengthen the rural health workforce (1). Evidence suggests that early rural exposure positively influences health professionals' decisions to practice in rural environments (2). Because clinical exposure in a rural setting may better capacitate and retain the future rural workforce (3), this contextual educational approach has intuitive appeal for countries where the rural health workforce remains challenged by inadequate resources. Much of the positive evidence regarding the efficacy of educational strategies to address rural health workforce needs has been generated in high income countries (HICs)—Australia, USA and Canada (4–6). While there is growing evidence for efficacy in other contexts (7–10), comparatively little is known about the effectiveness of these strategies in Low and Middle-Income Countries (LMICs) despite these contexts being most affected by rural health workforce shortages (11). Considerable differences in the healthcare climates between HICs and LMICs could plausibly impact the efficacy of rural training pathway strategies across these contexts. If LMICs are going to use undergraduate rural clinical exposure as a strategy for rural pathway development (12), it is critical to understand what the barriers to and facilitators of developing training sites might be. The need to rethink how the future workforce is trained, where they are trained and how to support existing rural health care services is imperative in speaking to the needs of LMICs and countries where disparities in health care provision are significant (2, 13), especially because of the complexity involved in implementing such strategies given the demands of competing healthcare priorities (12).

The classification of LMICs is based on the Gross National Income per capita, which categorizes South Africa (SA) as an upper-middle-income-country (11). One could therefore argue that experiences or research relating to health care in SA may not be comparable or transferable to LMIC contexts. However, SA is highly disparate, with the highest GINI coefficient worldwide, which impacts all of society, including the healthcare system. Healthcare disparities exist between the private and public healthcare systems and geographically between urban and rural locations (14). Rural, public healthcare facilities in South Africa face many of the same challenges that are prevalent in LMICs. Inequitable resource allocation, inadequate infrastructure, and severe personnel shortages are but some of the major health system challenges (15). Approximately 83.5% of SA's population are dependent on the public health care system (16) which is staffed by 30% of the medical personnel (17). There are stark disparities in specialist distribution in the public and private sector with 7 specialists per 100,000 population in the public sector compared to 69 in the private sector (18).

There is emerging evidence that undergraduate distributed clinical training for final year health professional students can complement quality of care and help alleviate clinical workload in rural and resource constrained environments (8). Students are perceived to improve patient satisfaction, workforce competency development and community-based services in other rural and urban SA contexts (8). By improving quality of care and potentially the accessibility of services, partnerships between rural health services and universities have the potential to meet some of the universal health coverage goals despite the current human resource challenges (19). Locating undergraduate health professions training outside of urban areas is a necessary intervention in the SA context to prepare the future health care workforce of the country (20). In order to deliver undergraduate clinical training successfully at rural, distributed sites in the SA context, major health system challenges such as the lack of physical teaching spaces, the burden of heavy workloads and the shortages of both academics and clinicians must be overcome (21).

The latent potential that exists within a health system-university partnership speaks directly to the need for a cooperative approach in facing the health challenges that exist at district level across SA (22). Ensuring sustainability of a distributed training site is necessary to optimize the potential value of university-health service partnerships. Based on a review of 19 studies, Scheirer (23) presents five crucial factors that influence the degree of sustainability of health-related programs, which provide a useful framework for assessing the sustainability of a clinical training site. These factors are (a) being able to modify the project to suit the local environment; (b) having a champion at the project site; (c) ensuring the project is aligned with the organization's mission and vision; (d) visible benefits to clients; and (e) support from stakeholders in other organizations (23).

### Contextual Background

The Stellenbosch University (SU) Ukwanda Centre for Rural Health (Ukwanda) has developed a network of rural distributed training sites to support undergraduate rural health professions education since 2002 and currently manages programmes across 5 rural towns within the Western Cape. The development of these distributed sites was slow and deliberate, spanning over 10 years and relying on established relationships within a relatively well-resourced rural healthcare system. In 2018, Ukwanda expanded SU's rural distributed training footprint to the remote setting of Upington, situated in the Northern Cape Province, 9 hours drive from the SU Faculty of Medicine and Health Sciences campus.

The Northern Cape Province, with an estimated 1.29 million inhabitants, is the least populous but largest in South Africa. It has no medical school and faces significant health system challenges, which are akin to the health systems challenges in LMICs. Upington is home to the only regional hospital in the province, Dr. Harry Surtie (DHS) Hospital, with 327 beds, situated 400 km from its nearest referral hospital. The local health district is plagued by severe health personnel shortages. Locally there are 23.0 medical practitioners, 0.4 medical specialists, 5.5 physiotherapists and 4.0 occupational therapists per 100,000 population (24). Issues with recruitment and retention of health personnel have contributed to inefficiencies within the public health system in the province (17). In a 2020 publication investigating the technical efficiency of provincial public health care in South Africa, the Northern Cape Province was classified as inefficient in providing public health care and recommendations for recruiting and retaining more medical personnel, specialists and researchers were suggested by the authors (17).

These health system challenges and the geographical remoteness as well as the absence of pre-existing relationships between Stellenbosch University and the health service formed the backdrop for the development of this clinical training site.

A process of cooperative development between the Northern Cape Department of Health and Ukwanda resulted in four final year medical students pioneering a longitudinal integrated clerkship of 10 months at DHS Hospital in 2019. Additionally, final year students from medicine, occupational therapy and physiotherapy undertook shorter rotations of between 4 and 6 weeks over the course of the same year. During this initial phase of development all students were primarily based at the hospital and clinical exposure varied across programmes with students either completing traditional discipline-based rotations or integrated rotations across both inpatient and outpatient settings. In addition, medical students rotated through four local primary care clinics in the surrounding area.

Local clinicians fulfilled dual roles as health care providers and student preceptors. Clinicians were supported in their roles as clinical educators through outreach visits and faculty development workshops conducted by academic staff from SU. Supplementary academic support was provided by SU and varied across programmes, including direct contact or online support or both. Online support activities included tutorials, case management discussions and remote ward rounds to supplement students' learning.

The rate and scale of this development and its interprofessional ethos against a backdrop of geographical remoteness and significant health system challenges has been encouraging. Understanding if an over-burdened health system in an under-resourced context is able to offer sufficient support for sustainable undergraduate health professions education has potentially important implications for rural workforce development in LMICs. However, exploring mutual benefit and the potential this initiative may have on the existing rural workforce is equally important.

The aim of this paper is to explore the potential value of this educational strategy for promoting rural workforce development from the perspective of multiple stakeholders. The degree of sustainability of this clinical training initiative is explored using the five factors recommended by Scheirer (23).

## Methods

Ethical approval was obtained from Stellenbosch University's Faculty of Medicine and Health Sciences Human Research Ethics Committee (#N19/02/026) and permission to conduct the research was provided by the Northern Cape Department of Health and Stellenbosch University Committee for Undergraduate Teaching. Only individuals providing written consent were included as research participants.

A case study design using an interpretivist paradigm was adopted for this study. The system under scrutiny was the training site as a whole. The study population comprised of students, student supervisors, academic programme and facility managers from the clinical and academic sites, and nursing preceptors, district managers and local clinicians working at the Hospital. Fifty five participants (Table 1) participated in various activities between January and November of 2019 to explore perceptions related to the value of the expansion initiative and the degree of sustainability (25). Triangulation of data collection and analysis contributed to the trustworthiness of the data and credibility of the findings (26).

**Table 1:** Summary of participants.

**Participants**	**Number of participants**	**Data collection: method**	**Data collection: person**	**Abbreviation used in article**
Supervising clinicians	6	Semi-structured individual interviews^*^	Research Team	SC
Facility Managers	3		Research Team	HFM
Academic Programme Managers	8		Independent researcher	APM
Longitudinal Integrated Clerkship Medical Students	4		Research Team	MS
District Health Managers	5	Focus group meeting^*^	Research Team	FGDM
Nursing Preceptors	3	Focus group meeting^*^	Research Team	FGNP
Non-supervising clinicians and staff	12	Brief semi-structured conversations^*^	Research Team and assistant familiar with hospital	NCS
Short Rotation students	14	Pre- and post-rotation surveys^**^	Research Team	SRS
**Total participants**	**55**			

* Interview schedule Appendix 1.

** *Interview schedule Appendix 2*.

Qualitative data was collected using semi-structured individual interviews, focus group interviews, brief semi-structured conversations and open-ended survey questions. Interview guides (Appendix 1) were used for all interviews and aimed to explore stakeholders' perceptions and future aspirations related to the expansion of the clinical training site.

### Semi-structured Face-to-Face Interviews

Purposive sampling was employed to select 21 participants for individual interviews (Table 1). Two participants who were crucial to the initial engagement with the Upington stakeholders were interviewed twice—at the beginning of the academic year, prior to the students' arrival and at the end of the academic year, prior to the students' final examinations. One of these participants' role expanded from a medical student supervisor to a newly appointed facility manager at the time of the second interview. An adapted interview guide exploring both roles was used (Appendix 1).

### Focus Group Interview and Management Meeting

Two focus group meetings were held with nursing preceptors and district health managers, respectively (Table 1). An adapted interview guide with several open-ended questions explored district health participants' expectations, concerns, opportunities and future aspirations regarding expansion of faculty programmes into the district clinics.

### Brief Semi-structured Conversations

Brief semi-structured conversations took place in the physical locations where the staff were working in order to use the environment as a prompt, by letting them envisage where and how students had engaged in that context (27). Twelve consenting staff members, from 5 hospital departments, namely the Rehabilitation, Internal Medicine, Pediatric, Orthopedic, and Surgical departments as well as staff working in the Intensive Care Unit participated. Participants included professional nurses, enrolled nurses, administrative ward clerks, newly qualified medical and allied health clinicians who worked and interacted with students but were not directly involved in their supervision. A research assistant, who had no prior relationship with any of the departments or staff working in the wards, conveniently selected individuals who were available and willing to participate at the time the researchers visited the ward.

### Survey Responses

Final year undergraduate students enrolled in Medicine (Primary Health Care module), Physiotherapy and Occupational Therapy undertook short clinical rotations for between 4 and 6 weeks. Two surveys were developed (Appendix 2) and distributed to the 14 consenting students through the Research Electronic Data Capture (REDCap^TM^) software (28). Pre-rotation surveys were sent prior to the start of students' rotations. The survey explored their expectations and concerns about the upcoming rotation. Post-rotation surveys were sent on the day that the rotation ended. The survey documented their experiences, and comments on various aspects such as supervision, interprofessional engagement and clinical exposure.

### Analysis

All semi-structured interviews, focus group interviews, and brief conversations were audio-recorded using a digital recorder, transcribed verbatim and translated from Afrikaans to English where required. All data was de-identified by assigning pseudonyms describing participants' roles (Table 1). Transcripts were emailed to all individual interview participants for member checking; five were returned with edits. Inductive analysis was initially undertaken by five members of the research team who immersed themselves in the data and developed individual code lists, before combining these and agreeing on a final list of codes for detailed analysis. All transcript and survey data were then allocated to members of the full research team, who undertook further detailed thematic content analysis using the code list.

## Findings

The analysis of the findings identified five main themes representative of the participating stakeholders' perceptions of the University's engagement with the hospital in the development of a new clinical training site in Upington. See Table 2.

**Table 2:** Themes and sub-themes.

**Theme**	**Sub-theme**
Theme 1: The process of development	Communication Phased approach
Theme 2: Influence on the health service	Workload Specialist outreach Quality of care Accountability
Theme 3: Workforce sustainability	Professional development and incentives Sense of purpose and value
Theme 4: A change in perspective	The value of training on the distributed platform Faculty development for distance learning
Theme 5: Equipping a future workforce	Further expansion Potential influence on district health services Relevant and accessible training Rural pathways

### Theme 1: The Process of Development

The process followed in developing the training site was seen to be an important contributor to the early successes that were spoken of by participants.

The “teaching institution and practical institution. We need to work together. We need to see through the same glasses. Because we have one vision.” (HFM 3).

The partnership that developed between the University and the local health service and the need for continuous communication during the phasing in of students at the site required persistent engagement between people from both institutions.

“I thought these people will never come back because in the beginning it was such a struggle—people didn't want to become involved” (SC 5).

This presence and interaction provided the space for people to be more open to partnership development

“Once we have had contact sessions with you guys [from Stellenbosch University] as well and we have done the training and stuff I think they have kind of seen how valuable the idea is as well so everybody is much more positive and they really want to engage in the things that we do.” (SC 5).

Provision was made to support students academically and in terms of logistics with the construction of a learning center equipped with the necessary information and communication technology, which afforded further partnership development. The learning center

“will benefit other people as well if they should have a need to but where students can actually go to a dedicated learning center and access new learning materials etc. So that is something new that is coming, and it is specifically related to this site.” (HFM 1).

#### Communication

Communication, which was perceived to be open and accessible, was recognized as an important factor for the initial and sustained development across all elements of the partnership, such as academic, logistic and managerial aspects in terms of student training, University and hospital policies, and the comings and goings of students and staff:

“so it's nice if there could be open communication the whole time because I don't think there will be something that we can say now already that this is going to be a problem, but as we pick up stuff we have to have open communication the whole time.” (SC 5)

Clear communication channels, crucial for effective communication between multiple stakeholders, were not always maintained. The need to keep all parties informed was evident especially when it related to students' academic exposure.

“The guidance we received because they have that little booklet with all the instructions and the rubrics and all those things so we know the students know exactly what they supposed to do and even how the assessment is being done so the guidance through that wasn't a problem.” (HFM 1)

Although some clinical supervisors at DHS Hospital felt that communication regarding students' learning activities and the expectations of the University was helpful in preparing them as clinical teachers, this was not the case for all academic departments.

“The communication channels particularly down to us because the students will have the information sometimes. But then we don't have the information. I think that we should also be included.” (SC 6)

Having an on-site student support person, someone who is not responsible for academic supervision, was important not just for students, but also to alleviate the burden on supervising clinicians.

“Supervision from the Ukwanda side for student well-being was really good. The speech therapist [X] was very *oppit* [with it] and always available for us to air concerns and responded to us very quickly and always kept us in the loop of what was happening and when things were happening.” (SRS 4)

Communication relating to specific policies and procedures for student safety was perceived as inadequate:

“Issues like what happens when they get sick, needle prick injuries what is the policies around certain aspects of the programme. Who is responsible if somebody get injured here? who do we phone, who do we report it to? You know that kind of thing that is not very clear.” (HFM1)

Miscommunication and lack of communication could be mitigated by having a go-to person, which promoted improved communication between academic departments and supervising clinicians:

“I know that [X] has got an open line, so he's quite happy to phone me. Uh, we've actually just spoken before this call. … so, I'm hoping that we are supporting, um, him and his team adequately.” (HFM 10).

Some academic departments adapted their approach to address challenges with communication by using information technology apps to support students and clinicians:

“We started a little WhatsApp group and said, ‘You know just ask if you don't know’. … we established open communication with them to say, ‘Your initial case studies; send it onto us if you are unsure if what you are feeding back is the correct thing to feedback to the students.’ So ya we kept it very informal, but within a very robust, existing structure, if that makes sense?” (APM 8)

#### Phased Approach

University and health service staff, alike, perceived benefits to gradually phasing in student involvement at the training site, which allowed time for clinical staff to adjust to the demands of clinical teaching and to feel better prepared.

“‘How are we going to supervise the students and do your own job?’ and then they gave training and everybody realized that you don't have to be with every student every hour of the day and everybody started thinking a lot about it.” (SC 5).

“I must say initially I was positive, but hesitant because you know you always think, ‘It's normal’ and I was wondering about the various departments and their lack of staffing etc. and will the students get a good academic, but I think at that point I wasn't 100% sure of what the programme was all about and even when I learned about it, it was still difficult to wrap my head around it, but now that I completely and fully understand it I am all on board.” (SC 6)

Gradual phasing in of the students allowed for quality control measures to be implemented:

“I like the idea of starting slowly, making sure that things are in place before we send students to a specific department or discipline in Upington where we are not sure what the quality of training is going to be.” (APM 1)

### Theme 2: Influence on the Health Service

The development of the clinical training site and the presence of the University in Upington was perceived to have an influence on the health care service, specifically influencing the workload of staff and the quality of care patients receive. Students were

“extra help for the doctors … it helps them a lot. Some of the doctors get stuck, sometimes we are one or two doctors short and then it helps when they are in the ward.” (NCS 8)

#### Workload

“You see them discussing patients with the senior doctors and then they help with the drips and taking blood.” (NCS 1)

The influence of the students' presence at the hospital on workload was mostly positive, with time taken engaging with students in the working environment providing opportunities for staff to interact differently with their patient loads.

Challenges in terms of time spent with students related mostly to administrative and mentoring activities, which were not necessarily part of everyday practice, but this balanced out further into the students' training when they were able to work more independently:

“I must say the administration part and having to mentor them, it does take some time. So, I must say uh yes, we are a bit pressed for time here to get all the stuff done that we have to get done. I think we are short of staff, so I think that's also contributing to the problem… But then after that when they start to you know work more independently, they see some of these patients alone. So that does mean I had to see them anyway, so then that takes a bit of the patient load in the ward off of our backs.” (SC 4)

In some instances, having students train at the hospital enabled additional services to be offered:

“So, it is not just that somebody is going to take up all your time and you have to be there 24/7. It's going to be a bit more that we can reach patients that we wouldn't have maybe been able to reach otherwise.” (SC 5)

For example

“with the psychiatric patients they were very helpful … because … some of our doctors don't want to deal with the psychiatric patients.” (NCS 8)

#### Specialist Outreach

Having the University engage with the hospital enabled specialist support for departments because of specific awards received from the Discovery Foundation to support specialist visits as well as specialist visits from Tygerberg academic departments every month to support student training.

“Other programmes that started as a result of the partnership, for instance doctors that are coming in, the Discovery grant issue that pay for [a private internist's] visits so those things wouldn't have been initiated if the students weren't here.” (HFM 1)

“Their support from the doctors that have come from Tygerberg. This is something good.” (SC 1)

However, not all clinicians could benefit from the perceived value that visiting specialists added because of their existing workload.

“I normally only made one round on that day with them because I was very busy. It was a very bad thing because I wanted to be there all 3 days because then I know more or less what they were demanding.” (SC 1)

#### Quality of Care

Having students train at DHS Hospital was seen to have a positive influence on the quality of care provided to the patients in the hospital. The thoroughness of the students' consultations also offered a more holistic understanding of patients' challenges and context, saving the clinician time. This was also recognized by staff not directly involved with student supervision.

“The social part of it … so most of the time we get that information from the student because they've taken [a] thorough history on the social impact of the child's life, uhm, so they go beyond the medical part of it to see what is wrong with the child maybe there's something wrong in the family that is causing the patient to be ill.” (NCS 4).

This was perceived to be as a result of the time students spend with patients and the effort they put into their work. Improved quality of care was also seen to arise from the perceived increase in patient satisfaction, because

“sometimes the patient does not feel comfortable talking to the doctor … and this is where the students comes in … they give them that extra hearing, they lend an ear when like the doctor is only concerned about the wound and don't address the fears” (NCS 3).

“I feel like [having the students] it's improved service delivery” (SC 5)

“because the students have to be so on top of it, they have to be so perfect and diligent in what they do, that they give the best care that they can for the patients” (NCS6),

Their enthusiasm also drives them, so that

“they are eager to see what new implementations they can implement in order to improve the service the quality of service we are rendering.” (NCS 5)

The thoroughness the students present in their work was also seen to be an important part of the quality of care offered.

“Their notes and so, they are very thorough. They do it the way it should be done.” (NCS 8)

Students' contributions were described in terms of specific areas of input and assistance that went beyond dealing only with patients. Projects, research and different perspectives were all seen as valuable. An example of this was the quality improvement project undertaken by medical students:

“The little projects that the students do, you know, the quality projects, those projects also change certain dynamics within the hospital for instance the [medical] students were doing a project on triage systems. I mean that has a big impact on the way nurses and doctors have to function in the ER [emergency room] unit now.” (HFM 1)

Projects students engaged in incorporated reviewing processes to improve patient satisfaction.

“If you [go] down to the hospital you will see one of the projects it was received so well [by] patients and everybody so the students contribute to the hospital and the dynamics of the hospital.” (HFM 1)

#### Accountability

Clinical staff in DHS Hospital were driven to develop their own skills and knowledge in order to provide the best possible training to students:

“So, it forced me, at a personal level, to develop my skills, to study more, to upgrade my own knowledge space and I think that is exactly the same thing that it brought to the hospital. The doctors are now dealing with students that ask questions and it also forces them to spend time thinking about patients more than they would do normally.” (PFM 1)

“Because when they go with to a patient I need to do everything perfectly and to the T, which is the way we should always do it, but it's good to have the students here to just keep you on your toes around patients and to keep you up to date. When they are asking you questions, you have to be able to answer them, so you have to do your own research.” (NCS 6).

Some clinicians commented that the students' approach to patient care highlighted aspects they as clinicians may have lost along the way.

“At least the patients feel like someone is listening to them. And I think most of the time we all forget to communicate with the patient because we are all so focussed on how sick the patient is, and we forget that that person is a human with feelings.” (NCS 3)

The students' presence at the hospital resulted in self-reflection of the clinical staff and a desire and drive to want to optimize their professional and clinical practices. This was not only evident at a personal level, but at a hospital systems level as well.

Having students conduct quality improvement projects as part of their training at the hospital was seen to be beneficial in terms of the accountability that was inadvertently imposed on the hospital by these projects:

“Because there is a project that the students put up and the students evaluate the project. So inadvertently they are evaluating us. That is the problem. There are now eyes and ears that were not here before they pick up things and they ask why you doing that? Forcing you to think about things and what you doing but also [saying] ‘I don't think that thing was done correctly, the guideline says this’ so it not only forces you to think about it but follow protocols that you have put in place and people have really not followed.” (HFM 1)

### Theme 3: Workforce Sustainability

Establishing a clinical training site at DHS Hospital also appeared to influence the local hospital staff in relation to professional development and job satisfaction and in this way also workforce sustainability.

#### Professional Development and Incentives

“My expectation I think was from the onset that the involvement of the faculty would bring opportunities first of all, opportunities of learning. Because we didn't look for money as a way of compensation but what I get is access to study material, access to a library things like that, that for me is crucial you know and in monetary terms it actually translates into monetary terms because if I have subscribe to a journal it costs me some money.” (HFM 1)

Certain learning opportunities became available to staff based at DHS Hospital with the development of the partnership, such as free access to the University's scientific library database, continuing professional development (CPD) opportunities and the possibility of postgraduate study.

“So when I have a professor coming from the faculty coming for the students, I can set up a teaching session with the doctors as a CPD activity and already I have a specialist in the field so the cost factor is reduced because I don't have to fly in somebody and pay for accommodation and all that which is usually the biggest cost in CPD activities.” (HFM 1)

These professional development opportunities relate to potential improved staff retention and rural pathway development, which were not available before the partnership development.

“Doctors that want to specialize won't come here because it won't count anything toward the possibility of going and specialize so they will go to a bigger center where there is a possibility that they can get into a programme but if we can set up that kind of thing, it is an incentive for attracting more doctors to this side.” (HFM 1)

Evidence of this was already visible with clinicians in Upington engaging in professional development opportunities:

“Me and Dr [X] from [XX Academic department] she is helping me … I have to write up cases and we do Skype interviews so she helps me, and we discuss it … because of her I will be able to do my diploma next year.” (NCS 7)

“It will benefit some of the professionals here as well like if they want to go into some research or do a Masters or do something else to have that open door with the University as well.” (SC 5)

The development of DHS Hospital as a training site is seen to have the potential to influence the future workforce of the hospital, and of the surrounding facilities that refer to it. There is a local vision to

“strengthen the site to the extent that we can accommodate more students in particular programmes which in the end, if the programme is running very well might attract future doctors to come to the hospital because now they know there is this association with the University of Stellenbosch.” (HFM 1)

#### Sense of Purpose and Value

Having students at Upington offers a sense of purpose to the staff at DHS Hospital, who now felt responsible to teach a new generation of health professionals.

“I want to teach and I really also feel like because of the impact that my mentors had in making me a [XX] I am hoping to have a similar effect on a student, maybe not to be a [specific speciality], but to be a certain type of doctor, and students are also making me want to be better.” (SC 6)

The process of site development

“boosts the morale of not only students but also the people working here to see these people, academics from the University coming down here and spending time with us.” (HFM 1)

The engagement encouraged clinicians at DHS Hospital to value not only themselves, but also clinicians from other professional teams, promoting collaboration around patient care:

“I definitely think people are more attuned to people from other disciplines. So, it's like they're seeing everyone in a different light now that they have to be trainers and teachers. So, I think people are more open to teaching other people about their profession and the work they're doing. And also involving everybody in decisions being made. So, I think it's been a very positive experience.” (SC 5)

### Theme 4: A Change in Perspective

Despite initial concerns from students and Faculty staff regarding the adequacy of clinical training on the distributed platform there emerged a clear change in perception and a recognition of the potential value distributed training has in preparing a future workforce.

#### The Value of Training on the Distributed Platform

The notion of expanding the clinical training platform was daunting to some and it raised concerns about an increase in teaching load

“I am aware of the fact that the Department of [X] is very straining in terms of the teaching responsibilities and to add something more, which is 800 km away without the necessary support there, that's really my concern.” (APM 6)

However, other staff saw the partnership and expansion of the clinical platform as having potential to not just lighten the academic workload for Tygerberg staff, but also improve the quality of training students were receiving:

“A secondary advantage is lessening the student burden on the platform at Tygerberg. … we all know that the current load on the platform at Tygerberg is too heavy. You can't have training with thirty students around a patient's bed. So, the more we can send students out, the less we have problems with over supply of students on the Tygerberg platform.” (APM 1).

“We can't train more doctors here [Tygerberg], than we are training at the moment and we can't train the ones that we are training to be any better than they are if we don't make radical changes to the way that we're training, to the place that we're training, to the exposure that we give them. And in the end if we don't embrace these changes, we are setting ourselves up for failure in the future.” (APM 4)

There were instances where academic programme managers questioned the value of training students away from centralized specialist care due to the limitations in the specialist exposure the students would get in that discipline:

“I went there together with a group of other clinicians from all the departments. And we saw a very nice hospital. And some of the functionality was a little bit challenged, but there were some disciplines that were doing really very well. And then others have not so … understaffed and under managed. And the exposure to certain cases was very limited. Most [X] cases were sent away to Kimberley [Specialist hospital 400 km away] and then also my impression was it wouldn't be a good place to train students in [X].” (APM 3)

But students commented that despite their own initial hesitancy toward learning away from the specialist academic hospital, from doctors who are not from their training institution, they realized that they are actually good doctors and saw the value of the training they were receiving:

“It's difficult but I think to try to get out of the mindset of, you need Tygerberg tuts, you need this consultant to tell you this, otherwise you won't be a good doctor, cause I've learned from doctors here that are from universities that aren't like the best in the country, but they good doctors. So, and I would trust them.” (MS2)

The change in perspective was also expressed by an academic coordinator from Tygerberg regarding her previous misconceptions about where students should be trained:

“I think in terms of learning for me, it's probably exceeded my expectations in terms of what the students learn there. I don't know what I was expecting, but I feel like I am doing them a disservice by saying that, because they should trust that people at the district level know what they are doing. Our expectation going forward is that we will have more than the one [block]. We would like to develop the other 4 or other 3 strands of our fourth-year placements within Upington as well. So, I think that's the expectation going forward. … there are over-arching ideas that all 4 of the placements will be there, eventually.” (APM 8)

Despite the challenges of understaffed departments and the concerns about clinical exposure, local supervising clinicians put it as follows:

“I think it's a good thing that they are here … they are exposed to equipped hospitals that have everything and everything runs smoothly, and then they come here and see that we don't have everything. Uh we're short on staff, we have to think out of the box.” (SC 3)

This was echoed by a Tygerberg counterpart:

“I think the students have experienced maybe the true South African circumstances, as far as healthcare goes.” (APM 4)

#### Faculty Development for Distance Learning

The expansion initiative was perceived to be a valuable testing ground for innovation and planning for a new MBChB curriculum that is currently being developed at the Faculty. Amongst other things, the renewed curriculum intends to provide early clinical exposure to the primary care platform and longitudinal distributed placements in the final year of training.

“I think it is helping us is with the development and the thinking of the new curriculum. Because many of the things like the long distance learning and teaching activities, how we support people at those facilities, all of those things will help us to plan the new curriculum” (APM 5)

“It has made us more aware of the need for electronic media and other blended online teaching methods. So, we've been doing lots of little bits of pieces, videoing this and that. And so on. But it is not, that was just interesting how we actually need to develop a whole online programme that would expose people to practical clinical material, how to examine a patient. That kind of thing.” (APM 3)

Innovations from placing students in Upington for clinical training had ripple effects into other University programmes:

“So, we are using Microsoft Teams for our postgraduate training. Whereas it all started with the Upington students.” (APM 5)

There was not only new learning about distance education, but also around distance assessment and the feasibility of continuing to have exam processes that rely on a ‘one size fits all’ approach.

“I think what we have been speaking about is the nature of our exam situation … we require an external examiner to be there and that … so, we are sitting with ideas around whether we should change the way in which we examine because of the practicalities thereof and the logistics. … we have a good exam process I don't see us changing that much, but what I think needs to happen is that we need to get support … to make our exam process feasible within a setting such as Upington.” (APM 8)

### Theme 5: Equipping a Future Workforce

An important aspect of continued engagement is exploring stakeholders' future vision for the Upington training site and the University's engagement with the Northern Cape. Arising from their experiences of the year spent in Upington, the medical students were very positive about the future possibilities and the potential the platform has to expand.

“It can really become a good academic site, because there's a big hospital, there's a lot of things if you think all the resources you need are here … I think it can be an amazing place to send students to cause you're going to work as an intern in the essence of you're gonna be like independent, you're going to think for yourself and you're gonna learn to reason and all those things, but you're also gonna have someone to check on you and make sure you're on the right track.” (MS 2)

#### Further Expansion

Dreams for further expansion included becoming an extension of a University and an example of a Northern Cape rural clinical school, which could model how rural training should be done. The benefit that this would bring more support was noted:

“I hope that this programme can continue you know and that eventually who knows we might have a satellite campus with accommodation and the proper support framework … who knows, the Northern Cape doesn't have a medical school so if we can have at least eventually a little support campus off site from the University whether it is affiliated to Sol Plaatjie University or Stellenbosch it's not really for me an issue as long as the support comes.” (HFM 1)

This expansion was envisioned by both the staff of DHS Hospital and the specialist academic coordinators at Tygerberg.

“I think to see it grow. And to see something similar developing in Upington that we have in Worcester and the rural clinical school in Worcester. So essentially, a second rural clinical school in Upington.” (APM 1)

“I'm hoping that Upington will go from strength to strength. And also, then become another model of how teaching can take place outside of the tertiary setting.” (APM 5)

It was noted that achieving the dream of developing Upington into a major training hub would require ongoing faculty development.

“I think there should be a faculty development programme for the staff in Upington with regular sessions related to for example clinical training methodologies, and especially clinical assessment. Because the problem is always that you, when you start using people for training that has not been involved in training for many years, for example, these people need to be capacitated in terms of educational techniques, assessment techniques, these sort of things.” (APM 1)

#### Potential Influence on District Health Services

Having the University invested in the site brought with it perceived opportunities for district level outreach, which had previously happened at a Provincial level, but had not been happening for some time. A district official saw the University's involvement in Upington as an opportunity to expand services to more remote communities,

“we have a very vast district … we would like the services to go to the most remote areas within the district and we as a department would be prepared to assist if it comes to a push for maybe transport so that they [the students] reach those very remote areas within the district because it is where we have a lot of challenges in terms of service delivery.” (FGDM)

It was mentioned, however, that this form of learning and service delivery had not been utilized to its full potential yet since the students had little interaction with the district health services during their time in Upington. They expressed a desire to get more involved in the local district services and community outreach not only to benefit their own learning, but also to help support the existing services available.

“I feel we the only interaction we had was with one district clinic that we have asked and arrange with our clinicians to attend. I feel the hospital was a bit ‘isolated’ from the community in a sense. I feel our programme can be broadened to include more district clinics. The district physio is a comm serve with a very high workload. We as students can definitely help in this regard by being more hands and helping to see more patients in a shorter period of time. There is definitely a need for community projects within the district, and many opportunities for possible community blocks which was confirmed by the current district physiotherapist.” (SRS 4)

#### Relevant and Accessible Training

The potential the Upington training site offers does not only include undergraduate health professions education, which stakeholders believe should be expanded in its scope, but also postgraduate training and research. The relevance and nature of the training was seen as a catalyst for envisioned programme and research opportunities across all academic domains.

“We are very, very excited about primary health care, district level care because we think that that prepares our students 100% for community service placements so we are very excited about that and for it to become the longitudinal placement like we have in Worcester and then I think continued involvement not only in clinical training, but undergraduate research and potentially post-graduate research as well within those environments because in my field at least we tend to be doing the research within the major cities like Cape Town or Pretoria or Johannesburg, but realizing that that's not rural enough in terms of health care service delivery within the public service. That's what we are excited about, I think as a continued involvement across all our domains.” (APM 8)

The expansion of training offered to accommodate postgraduate education was viewed as a means to support local specialist competency in rural areas.

“A MO [medical officer] will come here knowing that they can stay here for the year and write their primary's, do their first year at this hospital and then if it is even necessary, go to Tygerberg and complete their registrar time there.” (HFM3)

“Yes. I think I'm also hoping for more doctors you know, registrars to be sent to Upington, so then also our specialty departments can also be better.” (SC 4)

“Having rotating registrars, having rotating specialists, having registrars train here actually and also having some staff going to Stellenbosch University to be registrars, also having more visits by the visiting specialists.” (HFM4)

#### Rural Pathways

There is potential for the existing partnership to provide not only possibilities for training for existing clinicians but also reaching out to local people within the community to support rural pathways.

“The University can have a camp in Upington or in the Northern Cape to expose the [school going] students that otherwise don't get exposed to other health sciences because everyone wants to be a doctor, but they forget you can be a physio and an OT.” (HFM3)

“I hope that we can open more to the people of the Northern Cape, maybe more training like primary healthcare you know make it more accessible for people who want to study… from our province.” (FGDM)

“For the people of Upington and surrounding areas, our young generation if there is a possibility through the hospital or through the province to give them bursaries to go and study so that they can come back and plow back into their communities.” (HFM4)

The potential the partnership had for improving rural pathway development in the province by recruiting potential students from a young age was expressed:

“Because of our partnership say to the University say - Listen here are 20 students from poor backgrounds, but they are from Upington or they are in surrounds and they want to study Health Sciences - So it's sort of like a gateway for them.” (HFM3)

Some Faculty members envisioned the change that this approach to distributed training could have on the distribution of health care training in South Africa.

“I think the opportunity for increased exposure of our students to rural and semi-rural health care. And ja, I think in a, even more visionary sense perhaps. The improvement of quality of health care in Upington and surroundings. And, closer ties with the provincial government of the Northern Cape closer ties with especially the department of health of the Northern Cape Province. And then in strengthening those ties, the possibility of further expansion, of upgrading [the] platform in the Northern Cape. Not only in Upington.” (APM 1)

“Then I suppose from the hospital's point of view and the province at least my vision and I hope that, that is true for the rest of the province and the hospital as well is that by getting students to work in the peripheral regions because of staff issues that in future it might also prompt students to go, ‘Well, it's not so bad to work in a rural area or hopefully it's not so bad to work in Upington’ and maybe as a … medical officer that would then see the need to come and assist and help and work here.” (HFM3)

“Then with the students themselves … we are planting that seed of its okay to work here. Go off somewhere and post it, to their internship and then they apply to come back here for the year or for 2 years.” (SC 6)

## Discussion

Our study findings highlight the value added to existing health services, workforce sustainability and health professions education when establishing a distant training site for undergraduate students in a resource limited and over-stretched rural health care system. The mutual benefit derived through collaborative engagement of the University and the health care system, the importance of multi-level engagement in the development of a distributed training site and factors which ensure the sustainability of the project are explored.

The findings of this study support the emerging evidence that undergraduate distributed clinical training for final year health science students can complement quality of care and help alleviate clinical workload in rural and resource constrained environments (8). Students are perceived to improve patient satisfaction, workforce competency development and community-based services in other rural and urban South African contexts (8) and despite the rapid nature of the Upington training site development and the distance of the site from the academic training institution, these positive outcomes are still evident from this study. The perceived effect that not only students, but also the partnership between University and hospital have on the existing health service is notable and speaks to the reciprocity that one endeavors to achieve as a socially accountable training institution (29).

Although there is growing evidence that rural exposure during undergraduate training in LMICs influences rural pathways (2, 30), the findings of this study suggest that collaborative engagement between universities and the health system can influence future workforce development and rural retention. This can be achieved not only by enabling undergraduate rural training, but through continuous professional and competency development of local health service staff, with the potential for postgraduate training, both of which have been shown to influence doctors' decisions to remain working in rural areas (31). The perceived value of visiting specialists from the training institution not only to support student learning but also contribute to professional competency development has an influence on workforce sustainability. Partnerships between academia and health systems resulting in postgraduate training opportunities and career development can make long-term rural placements more attractive to young ambitious professionals wanting to specialize, which evidence suggests is one of the deciding factors in rural retention (1). This requires a sustained committed relationship between academic institutions and health services.

Opportunities for students and academic staff to experience and witness the challenges of working in rural underserved environments, made possible through the collaborative partnership between SU and DHS hospital, had a potentially transformative effect not just on students, but also on academic programme managers who are specialists in an urban tertiary level hospital. The findings of this study demonstrate a positive change in some faculty members' perceptions and understanding of the value not only of distributed clinical training, but also of rural health care and its challenges. This demonstrates improved insight into health in context and the importance of relevant curricula not just for students but for professionals as well. Considering academic outreach as a model for socially accountable engagement and rural exposure for academic relevance not just for students, but for academics as well is worth further exploration.

The concept of responsive adaptability, one of the key principles of establishing distributed training sites (32), served as a basis on which much of the Upington development took place and is believed to have encouraged sustainability of the initiative during the expansion of clinical training in Upington. The receptiveness of the broader community, but specifically the DHS staff in collaboratively creating a shared vision and strategy for each department was crucial and suggests this was not simply a theoretical notion, but a lived experience that was perceived by the stakeholders at a practical level, and accords with the literature on decentralized training (10). The findings of this study are consistent with the perception that the overall contribution students can make to a clinical environment is significantly more than the time invested in initial orientation and supervision, which is well-documented and evident in several contexts (33–35).

The value of ensuring sustainability of the rural training site was recognized by all stakeholders. Our data indicates that the development of the site was aligned with the five factors that influence the degree of sustainability of health-related programs identified by Scheirer (23). We have tabulated the perceptions of the stakeholders regarding the Upington expansion project in relation to the five factors influencing sustainability (See Table 3). Whether these initiatives are indeed sufficient to ensure sustainability of the newly developed site will need to be confirmed in follow up studies. However, there is no doubt that sustainability is a critical element in the successful implementation of rural pathways to train, develop and support health workers in LMICs (12), of which the Upington clinical training site represents an example.

**Table 3:** A summary of stakeholders' perspectives on the development of the Upington training site that relate to programme sustainability as described by Scheirer (23).

**Five factors influencing sustainability**	**Findings**
Being able to modify the project to suit the local environment	• Phased approach to engagement • Responsiveness and flexibility of learning activities • Aligning learning to DHS hospital needs • Gradual increase in student numbers • Intentional reflection on expansion project through a 5-year longitudinal study exploring the impact of site development
Having a champion at the project site	• Crucial identification and involvement of a local champion on site • Deliberate process of identifying champions at various levels within the health care system and Faculty • Clinician willingness to accommodate and train students at DHS Hospital
The project being aligned with the organization's mission and vision	• Collaboratively defining a joint mission and vision in the initial stages of development • Regular reflection on the process of engagement and the alignment thereof during the phased approach to engagement
Visible benefits to clients	• Interprofessional interactions at the facility promoting collaboration among departments • Improved staff morale • Perceived influence on quality of care and patient satisfaction due to student involvement • Increased access to reliable sources of information via University library • Opportunities for continuous professional development and the possibility of postgraduate programs • Contextual learning opportunities afforded to undergraduate students • New training opportunities at distributed clinical sites to accommodate the growing number of undergraduate students
Having support from stakeholders in other organizations	• External funding for visiting specialists to support service delivery in Upington • External funding awarded to support district level outreach and clinical training programs for staff • Stellenbosch University Network for Strengthening Rural Inter-Professional Education (SUNSTRIPE) engagement with continuous interprofessional and capacity development • Center for Health Professions Education faculty training workshops: train the trainer course

The findings of this study must be interpreted with caution. The data is limited to the perceptions of three key groups of stakeholders involved in the development of the Upington training site and does not include the perceptions of community organizations or patients and their families. However, every effort was made to include a wide range of participants with varied perspectives on this project.

## Conclusion

This paper presents evidence to consider the development of a new remote site for undergraduate clinical training as a strategy for developing rural pathways and influencing workforce sustainability in rural health systems. The challenges from this study are framed within the context of a stretched rural healthcare system, and the influence of this initiative on workforce sustainability and the contribution to rural pathways is explored. The process of site development, as perceived by the stakeholders may be transferable to other LMIC settings due to the challenging and resource constrained nature of the study environment. In particular, we have demonstrated the value of using a lens of health programme sustainability to review the process of learning site development. The need to rethink how and where the future workforce is trained, and how to support existing rural health care services through this process, is a critical element of developing sustainable rural training pathways in order to address the needs of LMICs and indeed all countries where inequity in health care provision is evident.

## Data Availability Statement

The datasets presented in this article are not readily available because they are still undergoing further analysis related to teaching and learning on the distributed training platform for the purposes of additional publications and feedback to stakeholders. Requests to access the datasets should be directed to Jana Muller (janamuller@sun.ac.za).

## Ethics Statement

The studies involving human participants were reviewed and approved by Stellenbosch University's Faculty of Medicine and Health Sciences Human Research Ethics Committee. The participants provided their written informed consent to participate in this study.

## Author Contributions

JM: lead author, involved in protocol development, data collection, analysis, and article write up. CR: primary co-author, involved in data management and analysis, and article writing. SH, JB, FC, and EdP: co-author involved in study from conception through to analysis and contribution to article. KD: co-author involved in data collection, data management and analysis, and contribution to article. IC: principal investigator and critical voice, involved in data analysis, and writing up of the final article. All authors contributed to the article and approved the submitted version.

## Conflict of Interest

The authors declare that the research was conducted in the absence of any commercial or financial relationships that could be construed as a potential conflict of interest.
