# Supplementary material for: Training for Transformation: Opportunities and Challenges for Health Workforce Sustainability in Developing a Remote Clinical Training Platform
Source: Front Public Health. 2021 Apr 20;9:601026. doi: 10.3389/fpubh.2021.601026 (PMC8093558; doi:10.3389/fpubh.2021.601026)
Supplement: Supplementary file 1 [file Data_Sheet_1.PDF]

Appendix 1: Interview Guides

1. Interview Guide for Interview Guide for Focus group interview: Nursing Preceptors and focus group meeting: District Health Managers

a. What are your expectations of the FMHS involvement in Upington?
b. How do you think this involvement will influence the community?
c. What concerns do you have about the expansion of training to Upington?
d. What measures do you think need to be in place to make the project a success?
e. What support do you need from our faculty?
f. What is your future vision for this initiative?

2. Interview Guide for Supervising Medical and Rehabilitation Clinicians

a. Please tell me about your position and responsibilities at your facility
   - How long have been in your current position?
   - What does your current role entail?
   - What is your involvement with the Stellenbosch students?

b. Describe how you think the students may have affected/impacted your work
   - Has it/ will it affect the quality of care provided at the facility?
   - Do you think there has been an impact on patients and the community by having students placed at the facility?

c. Did you feel adequately prepared to handle the students?
   - Have you received any written guidance?
   - Are you aware of the expectations and outcomes for the student's time at your facility?
   - Do you feel these are contextually relevant?
   - Have you been orientated to their program?
   - Are you aware of the support structures available to assist you in your role?

d. What are your hopes and expectations of the future involvement of the university with your health facility?
e. Is there anything you want to share about your expectations of students training in your facility/ward/area?

3. Interview Guide for Academic Programme Managers and Hospital Facility Managers

a. Please tell me about your position and responsibilities at your facilities
   - How long have been in your current position?
   - What does your current role entail?

b. Please tell me about your involvement with the expansion of the clinical training to the Dr Harry Surtie Hospital
   - What is your understanding of the goals of the Upington expansion?
   - Have you personally been involved with the students?

c. Prior to the FMHS involvement in Upington what were your expectations?
   - Have your expectations changed?

d. Do you think the involvement of the FMHS has influenced the hospital, staff and students?
   - How has the health service responded to the students?
   - Were there adjustments that needed to be made to accommodate the students?
   - Were these easily implementable?

e. What challenges have you experienced or observed as a result of this initiative?
f. What are your ongoing support needs?
g. What is your future vision for this initiative?

4. Adapted Guide for Supervising Medical Clinician/Dr Harry Surtie Hospital Facility Manager

a. Please tell me about your position and responsibilities at your facilities

- How long have been in your current position?
- What does your current role entail?

b. Please tell me about your involvement with the expansion of the clinical training to the Dr Harry Surtie Hospital

- What is your understanding of the goals of the Upington expansion?
- Have you personally been involved with the students?

c. Prior to the FMHS involvement in Upington what were your expectations?

- Have your expectations changed?

d. Do you think the involvement of the FMHS has influenced the hospital, staff and students?

  - How has the health service responded to the students?
  - Were there adjustments that needed to be made to accommodate the students?
  - Were these easily implementable?

e. What challenges have you experienced or observed as a result of this initiative?

f. What are your ongoing support needs?

g. What is your future vision for this initiative?

Regarding your role as a student supervisor:

h. How do you think the patients, or the community are affected by having students at the health facility?

i. How adequately prepared did you feel to handle the students when they were here?

- Did you receive any written guidance?
- Did you receive any written guidance?
- How supported did you feel?

j. Have any new initiatives started in family medicine that you think came as a result of the SU-DHSH partnership, please describe them?

5. Interview Guide for Longitudinal Integrated Clerkship Medical Students

a. Describe your experience training in Upington this year? What were some of the most memorable moments?

b. Is there something that stands out for you in terms of your development as a person and professional?

c. What advice would you give to new students coming to Upington? What feedback would you give to the university and the hospital?

d. How would you envision the Upington project moving forward in the years that come? What would you like to see happen?

6. Interview Guide for Brief Semi-structured Conversations: Non-supervising Clinicians and Staff

a. What is your role in the Hospital?

b. What students have you interacted with during this year?
c. What is your experience of students working in your domain?
   - Describe how the students affected your work?
   - Can you show me some examples or describe how the students were involved in your domain i.e. what students have been doing, where they have been working or patients they might have seen?
   - How do you and your team feel about working with students?
d. In what ways do you think the students affected the patients and the care provided in the hospital?

Appendix 2: Survey (adapted to include only open-ended questions)

Short Rotation: Pre-placement Survey

We would like to hear your expectations about your rotation in Upington. What you are looking forward to and what you have reservations about. Please complete the form below to help us better understand where we can help meet your expectations.

Site (e.g. de Aar): ______________________

Programme (e.g. Nursing): ______________________

Rotation (e.g. Obs and Gyn): _________________________

Date (e.g. May 2019):_____________________

Open questions about your rotation in Upington (Please write legibly)
16. What I am most looking forward to about this rotation is/are …
17. What I am least looking forward to about this rotation is/are …
18. My biggest concern/s about this rotation is/are …. (We welcome any details regarding your answers to specific questions in the survey above to better understand some of your concerns).
Thank you for taking time to complete this questionnaire.

Short Rotation: Post-placement Survey

Please complete the general feedback form below to help us optimise the lived experience offered to students at this distributed training site.

Site (e.g. de Aar): ____

Programme (e.g. Nursing): ____

Rotation (e.g. Obs and Gyn): ____

Date (e.g. May 2019): ________________________________

Open questions about your experience of being in Upington (Please write legibly)	
1. The best things about the experience are/were ……	
2. The most difficult thing/s about the experience was/were …	
3. What recommendations would you make to improve the experience for the next group of students?	
4. Any specific concerns related to the questions answered in the survey above that you would like to expand on?	
	We would welcome comments you may have on any of the following areas
The type of clinical exposure you experienced	
Academic input	

Student supervision	
Interprofessional engagement	
Community engagement	
Transport arrangements	
Residence	
Communication with university coordinator (at Tygerberg)	
Safety	
Social life	
5. Was there anyone who became a role model for you and thus contributed to your experience?<br>• Yes<br>• No	
6. If yes, who?	
7. And in what way was that important?	
Thank you for taking time to complete this questionnaire.	
